# Gum Arabic as novel anti-oxidant agent in sickle cell anemia, phase II trial

**DOI:** 10.1186/s12878-017-0075-y

**Published:** 2017-03-16

**Authors:** Lamis Kaddam, Imad Fadl-Elmula, Omer Ali Eisawi, Haydar Awad Abdelrazig, Mohammed Abdelraman Salih, Florian Lang, Amal M. Saeed

**Affiliations:** 1 Department of Physiology, Faculty of Medicine, Alneelain University, P.O. Box: 11121, Khartoum, 12702 Sudan; 2 Alneelain Research Centre Faculty of Medicine, Alneelain University, Khartoum, Sudan; 3 Department of Hematology Military Hospital, Khartoum, Sudan; 4 Department of Pediatrics Military Hospital, Khartoum, Sudan; 5 Department of Biochemistry, Faculty of Medicine, University of Khartoum, Khartoum, Sudan; 6 Department of Physiology, University of Tübingen, Tübingen, Germany; 7 Department of Physiology, Faculty of Medicine, University of Khartoum, Khartoum, Sudan

**Keywords:** Gum Arabic, Sickle, Anti-oxidant, Oxidative stress

## Abstract

**Background:**

Sickle cell anemia patients suffer from oxidative stress due to chronic inflammation and self-oxidation of sickle hemoglobin (Hb S). Chronic oxidative stress contributes to endothelial dysfunction, inflammation and multiple organ damage in sickle cell disease (SCD). Thus, antioxidant medication may favorably influence the disease. Gum Arabic (GA), edible, dried, gummy exudates from Acacia Senegal tree, has been claimed to act as an anti-oxidant and cytoprotective agent, protecting against experimental hepatic, renal and cardiac toxicities in rats. We hypothesized that regular intake of GA increases anti-oxidant capacity and reduce oxidative stress.

**Methods:**

Forty-seven patients (5–42 years) carrying hemoglobin SS were recruited. Patients received 30 g/day GA for 12 weeks. Total anti-oxidant capacity (TAC), malondialdehyde (MDA) and hydrogen peroxide (H_2_O_2_) levels were measured by spectrophotometric methods before and after GA intake. Complete blood count was measured by sysmex.

**Results:**

Gum Arabic significantly increased TAC level *P* < 0.001and decreased the oxidative markers MDA (*P* < 0.05) and H_2_O_2_ (*P* < 0.005).

**Conclusions:**

GA has potent anti- oxidative properties in sickle cell anemia. The anti-oxidant effect of GA may thus favorably influence the clinical condition of this and further diseases characterized by oxidative stress.

**Trial registration:**

ClinicalTrials.gov Identifier: NCT02467257. Registered 3rd June 2015. Retrospective registration.

## Background

Chronic inflammation with oxidative stress emerged as an important pathogenic mechanism in sickle cell disease (SCD) [1–3]. SCD is primarily a disorder of RBCs, which are a significant source of free radicals in biological systems [4]. Oxidative stress may thus contribute to the abnormalities that underlie the clinical course of SCD [4]. Oxidative stress is one of the factors that modulate the phenotypic expression of SCD [5]. Oxygen has the ability to form highly reactive metabolites such as superoxide anion radical (O2 ^−2^), hydrogen peroxide (H_2_O_2_), and hydroxyl radical (•OH). These reduced metabolites of oxygen are referred to as “reactive oxygen species ROS” [6]. Oxidative stress can damage specific molecular targets (lipids, proteins, carbohydrates etc.), resulting in cell dysfunction and/or death. Oxidative stress level increases during vaso-occlusive crises and acute chest pain [7]. Oxidative stress is not only linked to chronic inflammation, it also contributes to endothelial dysfunction [8].

Sickle cell anemia patients have high levels of oxidative stress markers and low levels of antioxidant capacity. In addition to oxidative stress SCD patients have lower plasma levels of the antioxidant vitamins (A, C and E), lower serum levels of zinc and significantly higher serum levels of copper in comparison to controls [9, 10]. Zinc deficiency with a copper excess may contribute to free radical production and oxidative damage [9]. Deficiency of antioxidant vitamins (A, C and E) could account for some of the observed manifestations of SCD such as increased susceptibility to infection and hemolysis [11]. Any medication that increases the antioxidant capacity is thus expected to favorably influence the clinical course of the disease. Gum Arabic (GA) is an edible, dried, gummy exudate from the stems and branches of Acacia Senegal and Acacia Seyal. Oral intake of GA has been shown to provide several health benefits [12], such as prebiotic effects [13]. GA significantly increases Bifidobacteria, Lactobacteria, and Bacteriodes in the gut [13]. GA is claimed to have anti-cancer [13], anti-malarial [14] and immune-modulatory effects [14, 15]. GA is considered to act as an anti-oxidant and cytoprotective agent [16] and it can protect against experimental hepatic, renal and cardiac toxicities in rats [19]. GA is assumed to be effective mainly due to strong anti-oxidant properties [17, 20, 21]; GA may enhance the activity of superoxide dismutase (SOD) in kidney [22]. Amino acids tyrosine, histidine and methionine seem to be responsible for the antioxidant capacity of GA against ROS [23]. In experimental chronic renal failure (CRF) in rats GA administration decreased the superoxide production to control levels and raised the level of GSH and TAC. Alyahia et al. revealed that GA offers protection against cyclophosphamide-induced urinary bladder cytotoxicity in a rat model by neutralizing ROS and mitigating oxidative stress [16]. GA was effective as a potent superoxide scavenger in doxorubicin induced cardiotoxicity murine model [24]. Moreover, GA was found to decrease MDA renal level on Cisplatin-Induced nephrotoxicity rat model [25].

These observations suggest that GA may find clinical application in a variety of conditions where cellular damage is a consequence of oxidative stress like sickle cell anemia. We hypothesized that regular intake of GA would increase the TAC and decrease the oxidative stress markers. The present study tested whether Gum Arabic may have anti-oxidant properties in SCA patients.

To the best of our knowledge this is the first study conducted to investigate the effect of oral administration of GA on anti-oxidant capacity in sickle cell anemia patients.

## Methods

The participants of this study were recruited from the out patients clinic of pediatric and adult hematology units in Military Hospital-Khartoum-Sudan. The Inclusion criteria were: patients homozygous for SCD (SS) as documented by Hemoglobin electrophoreses. Their age ranged between 5 and 50 years. The total number of participants recruited was 47. All medications and dosages had been stable for 2 weeks before study entry. Exclusion criteria: patients receiving blood transfusion within the last three months or admitted to the hospital within 2 weeks because of SCD-related events or crisis.

### Gum Arabic administration

GA in powder form is a 100% natural extract powder produced mechanically from the wildly grown Acacia Senegal tree with a particle size less than 210 μm. GA in powder form was provided from Dar Savanna Ltd., Khartoum, Sudan. Properties and composition of GA are listed elsewhere [26]. The daily dose was 30 g, given in one sachet to be consumed early morning dissolved in water for 12 weeks. The GA was provided to the participants every two weeks for three months (14 sachets per each visit). Empty sachets were retained every visit as indicator of compliance.

### Sample collection and colorimetric determination of TAC and oxidative markers

Blood sample was collected before administering GA and after 12 weeks as follows:

Two ml in EDTA container and two ml in plain container. The serum and plasma was separated by centrifugation at 3000 rpm for 15 min then aliquot into four eppendorf tubes and stored at −85 °C till final analysis. All blood samples were collected by certified nurses in Military Hospital.

Antioxidant capacity in the serum was determined by the reaction of antioxidants in the sample with a defined amount of exogenously added hydrogen peroxide (H_2_O_2_). The antioxidants in the sample eliminated a certain amount of the provided hydrogen peroxide. The residual H_**2**_O_**2**_was determined calorimetrically by an enzymatic reaction which included the conversion of 3,5,dichloro −2– hydroxybenzene sulfonate to a colored product [27].0.5 ml of H_2_O_2_ was added to 20 μl of serum and incubated for 5 min at 37 °C. Working reagent was added and immediately the absorbance of blank and sample were read against distilled water at 505 nm.

The malondialdehyde level was calculated by the thiobarbituric acid reactive species (TBARS) technique. This method is based on the reaction of malondialdehyde and other aldehydes, which are by products of membrane damage caused by ROS, with thiobarbituric acid (TBA) at low pH and high temperature forming a complex with maximum light absorption at 535 nm [28]. One ml of TBA was added to 200μl of serum, mixed well in the test tube and boiled in 95 °C boiling water bath for 30 min. The absorbance of sample against blank and standard against distilled water was read at 534 nm.

Hydrogen peroxide was determined in the plasma by the reaction of H_2_O_2_ with 3, 5-dichloro-2-hydroxybenzensulfonic (DHBS) acid and 4-aminophenazone (AAP) in the presence of peroxidase (HRP) to form a chromophore. This method had been developed by Fossati et al. [29]. 500 μl of DHBS and 500 μl of AAP was added to 50 μl of plasma in a glass tube, and incubated for 10 min at 37 °C. The sample and standard were read against blank at 510 nm.

Complete blood count was measured using automated analyzer (Sysmex).

Data were analyzed using SPSS version 20. Paired samples T test was used to compare between pre and post intervention results. Person correlation was used to find correlation between different parameters. *P* values equal or less than 0.05 was considered significant.

## Results

Forty seven patients were enrolled (Table 1). All were Sudanese; 23 were males, age 5 to 42 years. Duration of treatment was for 12 weeks except two patients received GA for nine weeks and eight patients for ten weeks. The last recorded results were considered for final analysis as post treatment results.

**Table 1:** Demographics and baseline characteristics

Characteristics	Mean	SD	Median	Maximum	Minimum
Age	16.26	8.52	15	42	5
Gender	23(49%) Male				
Base line weight (Kg)	35 · 96	14	37 · 3	63	13
Base line height (Cm)	148 · 34	20.99	154 · 5	107	190
Hb g/dL	7.28	1.105	7	11	5 · 5
Hb F (%)	6.68	5.44	4.80	17.50	00
Hb S (%)	89 · 99	5 · 15	91	97 · 20	79 · 40
HbA_2_ (%)	3 · 33	0 · 52	3 · 3	4 · 4	2 · 5
TAC mmol/L	0.32	0.077	0.33	0.50	0.17
MDA nmol/mL	6.35	3.73	6.0	14.4	0.29
H_2_O_2_mmol/L	0.47	0.21	0.46	1.44	0.15

**Fig. 1:**
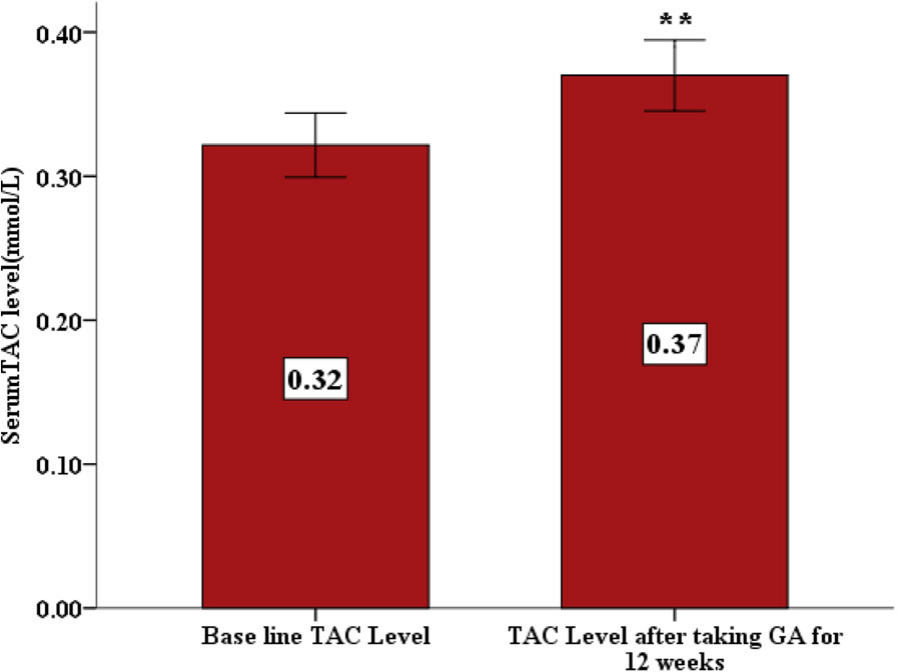
Effect of GA intake on TAC Level (*P* < 0.001)

**Fig. 2:**
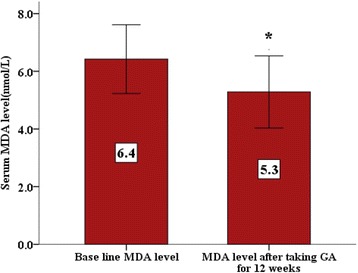
Effect of GA intake on MDA Level (*P* < 0.05)

**Fig. 3:**
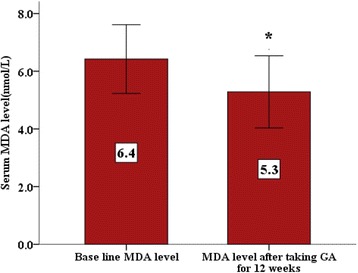
Effect of GA intake on H_2_O_2_ Level (*P* < 0.005

**Fig. 4:**
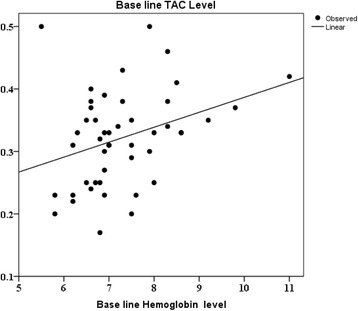
Linear regression between TAC and Hb level (*r* ^2^ = 0.109, *P* < 0.05). Dependent Variable: Base line hemoglobin level. Predictors: (Constant), Base line TAC level

**Fig. 5:**
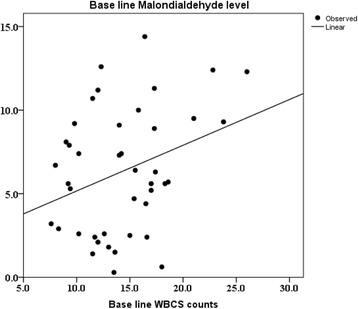
Linear regression between MDA and WBCs count (*r* ^2^ = 0.102, *P* < 0.05). Dependent Variable: Base line MDA level. Predictors: (Constant), Base line WBCs level

**Fig. 6:**
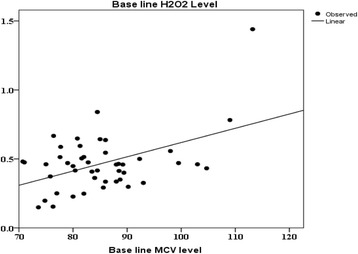
Linear regression between H_2_O_2_ and MCV level (*r* ^2^ = 0.219, *P* = 0.001). Dependent Variable: Base line H_2_O_2_ level. Predictors: (Constant), Base line MCV level

Oral Gum Arabic intake significantly increased the level of TAC (Fig. 1) and reduced the levels of both: MDA (Fig. 2) and H_2_O_2_ (Fig. 3). Response rate was 60%. We observed significant positive correlation between base line TAC and hemoglobin (Fig. 4). We also found significant correlation between MDA level and TWBC count (Fig. 5). H_2_O_2_ level was positively correlated with MCV baseline level (Fig. 6).

## Discussion

Sickle cell anemia patients suffer from oxidative stress [7] which is caused by chronic inflammation [1] and self-oxidation of Hb S [30]. Chronic oxidative stress contributes to endothelial dysfunction, inflammation and multiple organ damage in SCD [30]. Antioxidant enzymes were significantly less in red blood cells of SCA patients than in red blood cells of healthy controls [7]. Sickle erythrocytes have been shown to have elevated levels of ROS as compared to normal (AA) erythrocytes [1]. Chronic oxidative stress in SCD is caused by an imbalance between the production of reactive oxygen species (ROS) and antioxidant enzyme activity [8]

Agents that increase the antioxidant capacity of sickle patients were expected to improve their clinical condition [7]. Trials of antioxidant agents in mouse models of SCD also appear to reduce markers of acute and chronic inflammation [1].

In our study GA increased TAC of patients (*P* < .0.001). GA is known to have antioxidant properties, which have been illustrated in animal model of CRF [18, 31]. GA increased anti-oxidant enzymes like superoxide dismutase (SOD), catalase and glutathione [31]. GA microcapsules found to be potent anti-oxidant agent in vitro [23]. Our results confirmed that GA has anti-oxidative properties which can be utilized to improve patient’s condition and attenuate disease severity.

Oxidative stress, which is manifested by a significant increase in the levels of MDA and inhibition of the peroxidase and catalase enzymes, presents a major cause of the tissue damage [16]. MDA, which is a byproduct of lipid peroxidation [7], is higher in SCA patients than healthy controls [7].

GA significantly decreased MDA levels in a chronic renal failure (CRF) animal model and nephrotoxicity [25, 31]. In our study GA significantly decreased the MDA level (Fig. 2). This interesting result indicates that GA may have protective effect against oxidative stress and tissue damage in these patients.

Hydrogen peroxide (H_2_O_2_) was viewed as a toxic molecule to human tissues [32]. Sickle cell erythrocytes produce twice as much superoxide, H_2_O_2_ and hydroxyl radical as compared to normal healthy controls [10]. GA significantly decreased H_**2**_O_**2**_level in SCA patients (Fig. 3). Several studies pointed to catalase as the primary enzyme responsible for protecting the red cell from H_**2**_O_**2**_ [33]. GA increased catalase activity as evidenced in several animal models [31, 34]. GA also increases H_2_O_2_ scavenging capacity in vitro [23]. This may explain the decrease we have noticed in H_**2**_O_**2**_ level after taking GA for 12 weeks.

## Conclusions

Our results provided more evidence that GA has potent anti- oxidative effects in humans as demonstrated by its ability to increase TAC and to decrease oxidative stress markers in humans. All previous studies were conducted in animal models or in vitro studies. The present study thus uncovers a novel effect of GA which can be utilized in other clinical conditions and diseases caused by increased lipid peroxidation and tissue injury. Thus the increased intake of dietary antioxidants from GA may help to maintain an adequate antioxidant defense status and consequently contribute to the management of SCD.

## Abbreviations

GA: Gum Arabic
H_**2**_O_**2**_: Hydrogen peroxide
HbS: Sickle hemoglobin
MDA: Malondialdehyde
SCA: Sickle cell anemia
TAC: Total anti-oxidant
